# Evidence for phylogenetically and catabolically diverse active diazotrophs in deep-sea sediment

**DOI:** 10.1038/s41396-019-0584-8

**Published:** 2020-01-06

**Authors:** Bennett J. Kapili, Samuel E. Barnett, Daniel H. Buckley, Anne E. Dekas

**Affiliations:** 1 Department of Earth System Science, Stanford University, Stanford, CA 94305 USA; 2 School of Integrative Plant Science, Cornell University, Ithaca, NY 14853 USA

**Keywords:** Microbial ecology, Biogeochemistry, Microbial biooceanography, Stable isotope analysis, Environmental microbiology

## Abstract

Diazotrophic microorganisms regulate marine productivity by alleviating nitrogen limitation. However, we know little about the identity and activity of diazotrophs in deep-sea sediments, a habitat covering nearly two-thirds of the planet. Here, we identify candidate diazotrophs from Pacific Ocean sediments collected at 2893 m water depth using ^15^N-DNA stable isotope probing and a novel pipeline for *nifH* sequence analysis. Together, these approaches detect an unexpectedly diverse assemblage of active diazotrophs, including members of the *Acidobacteria*, *Firmicutes*, *Nitrospirae*, *Gammaproteobacteria*, and *Deltaproteobacteria. Deltaproteobacteria*, predominately members of the *Desulfobacterales* and *Desulfuromonadales*, are the most abundant diazotrophs detected, and display the most microdiversity of associated *nifH* sequences. Some of the detected lineages, including those within the *Acidobacteria*, have not previously been shown to fix nitrogen. The diazotrophs appear catabolically diverse, with the potential for using oxygen, nitrogen, iron, sulfur, and carbon as terminal electron acceptors. Therefore, benthic diazotrophy may persist throughout a range of geochemical conditions and provide a stable source of fixed nitrogen over geologic timescales. Our results suggest that nitrogen-fixing communities in deep-sea sediments are phylogenetically and catabolically diverse, and open a new line of inquiry into the ecology and biogeochemical impacts of deep-sea microorganisms.

## Introduction

Nitrogen is an essential element for life and microorganisms play a critical role in its bioavailability. Through the enzymatic reduction of dinitrogen gas to ammonia, nitrogen-fixing organisms (i.e., diazotrophs) produce the Earth’s largest natural source of bioavailable nitrogen [1]. In particular, marine diazotrophs supply nearly one-half of the global fixed nitrogen demand [2] and their activity often regulates marine primary productivity [3–5]. However, despite its well-documented ecological and biogeochemical importance in the pelagic euphotic zone [6], and its continued investigation in shallow marine sediments [7–10], nitrogen fixation in deep marine sediments (>200 m water depth) remains relatively unexplored. This may be a significant oversight, given that deep-sea sediments cover nearly two-thirds of Earth’s surface, have high microbial densities (up to 1000× greater than surface waters) [11], and can have relatively low concentrations of bioavailable nitrogen at the surface (<25 μM) [12].

Recently, nitrogen fixation has been detected or implicated in several geochemically anomalous deep-sea habitats, including methane seeps [12–15], hydrothermal vents [16], whale falls [12], and oxygen minimum zones [17, 18]. The emerging perspective from *nifH* sequencing [19], inhibition experiments [20], and geochemical correlation analyses [12, 15, 17, 18] suggests that the diazotrophs across these sites are phylogenetically and catabolically diverse. However, only two deep-sea taxa have been directly identified as diazotrophs to date, the methanogenic *Methanocaldococcus* sp. FS406-22 isolated from hydrothermal vent fluid [21] and the methanotrophic ANME-2 archaea found at methane seeps [22]. Even less is known about the identity of deep-sea diazotrophs outside these geochemically anomalous regions, despite diverse *nifH* sequences [19] and bulk ^15^N_2_ assimilation [12] suggesting their presence and activity. Identifying the organisms that fix nitrogen in widely representative deep-sea sediments would likely reveal novel nitrogen-fixing taxa, prescribe ecosystem function to uncultivated lineages, and help evaluate the biogeochemical impacts of and controls on diazotrophy in the greater marine benthos.

In the present study, we investigate the identity and activity of diazotrophs within deep-sea sediment collected at the distal end of Monterey Canyon, CA, USA (0–3 and 9–12 cm below seafloor [cmbsf]; 2893 m water depth). Active diazotrophy was previously demonstrated within this sediment via ^15^N_2_ tracer assays [12], however, the responsible organisms were unidentified. Here, we identify the active diazotrophs using a combination of density-gradient ^15^N-DNA stable isotope probing (^15^N-SIP) and a novel *nifH* amplicon analysis pipeline. We investigate sediment incubated with a headspace of either argon or methane to examine the effect of methane on diazotroph community composition and activity. From our integrative approach, we identify diazotrophs with broad phylogenetic and catabolic diversity, bearing implications for the resiliency and biogeochemical impacts of deep-sea diazotrophy through time.

## Materials and methods

### Sample collection and ^15^N_2_ sediment microcosm incubations

**Fig. 1:**
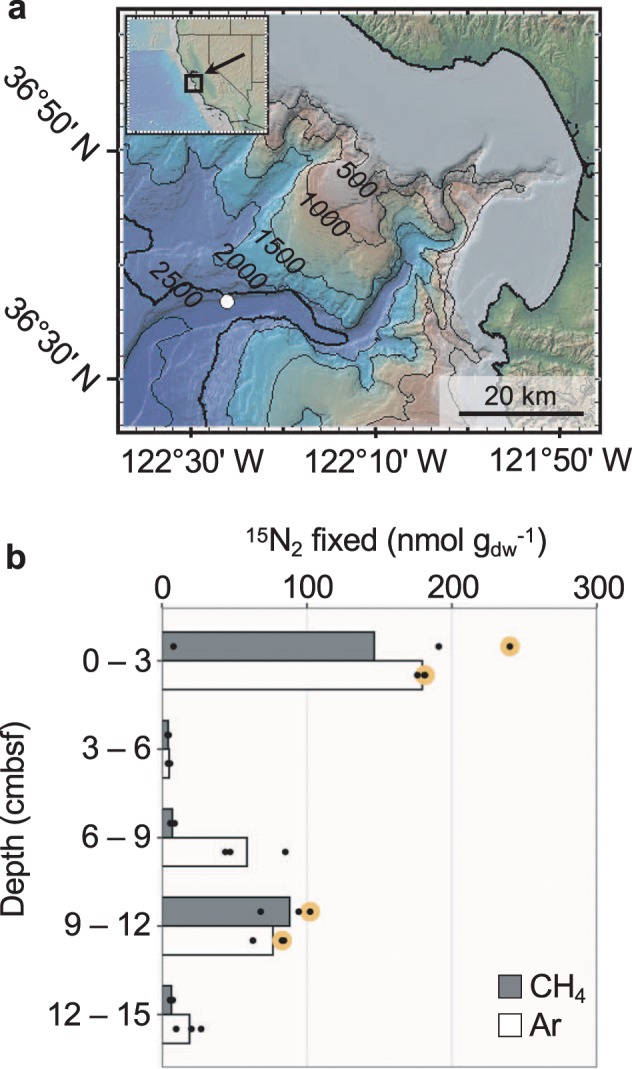
Sampling location and previously measured N_2_ fixation in the sediment core used in this study. **a** Map of sampling site at Monterey Canyon, California, USA. Sampled location marked with a white circle. Contour lines show 500 m depth intervals. **b** ^15^N_2_ assimilation in sediment microcosms measured using isotope ratio mass spectrometry (reproduced from Dekas et al. [12]). Microcosm headspace gas is indicated. Each circle represents a biological replicate, with bars indicating the average ^15^N_2_ assimilation. Large, yellow circles indicate the replicate bottle used for ^15^N-SIP and *nifH* analyses.

Sediment pushcores from Monterey Canyon, CA, USA were collected in October 2010 on the R/V *Western Flyer* using ROV *Doc Ricketts* (Fig. 1a). The sampling site was located at 2893 m water depth and showed no visible signs of physical or geochemical anomalies. The site was 28 m from a previous whale fall (deposited ~10 years prior to sampling). ^15^N_2_ incubations from the 0–3 and 9–12 cmbsf horizons were conducted with sediment mixed 1:1 with argon-sparged 0.2 μm-filtered bottom water in 60 ml serum bottles amended with argon or methane headspaces and ^15^N_2_ gas (Sigma-Isotec lot #SZ1694). For details on sample collection, sediment geochemistry, ^15^N_2_ incubation set up and subsampling, including an assessment of the negligible ^15^N-contaminants in the ^15^N_2_ gas, and bulk ^15^N-incorporation over time, see Dekas et al. [12].

### DNA extractions

DNA was extracted in duplicate from raw (unincubated) and ^15^N_2_-incubated (2-month time point) sediment using a PowerSoil DNA Isolation Kit (MoBio Laboratories, Carlsbad, CA, USA) following manufacturer protocol with the following modifications. Samples were initially centrifuged at 10 000 rcf for 30 s and supernatant removed. After addition of Solution C1, bead beat tubes were heated at 65 °C for 10 min, briefly vortexing after 5 min. Cells were lysed by bead-beating at 5.5 m s^−1^ for 45 s using a FastPrep-24 5 G sample homogenizer (MP Biomedicals, Santa Ana, CA, USA). Duplicate DNA extracts were combined and stored at −80 °C.

### Density-gradient stable isotope probing

Density-gradient separation of DNA was performed according to Buckley et al. [23] with the exception of excluding the secondary bis-benzimide CsCl gradient [24]. In summary, DNA fragments >4 kb were selected using a BluePippin platform (Sage Science, Beverly, MA, USA) and added to gradient buffer (15 mM Tris-HCl, pH 8.0; 15 mM EDTA; 15 mM KCl) containing 1.762 g ml^−1^ CsCl in 4.7 ml polypropylene tubes. Samples were ultracentrifuged to isopycnic equilibrium at 164 000 rcf for 66 h at 20 °C in a TLA-110 fixed angle rotor (Beckman Coulter, Brea, CA, USA). Following centrifugation, tubes were fractionated from bottom to top in 100 μl increments via displacement by Milli-Q water. Fraction densities were calculated using an AR200 Digital Refractometer (Reichert Technologies, Depew, NY, USA). Fractions were then desalted using an Ampure XP bead clean-up kit (Beckman Coulter) and stored at −80 °C.

### 16S rRNA gene and *nifH* amplification and sequencing

The 16S rRNA gene was amplified from all density fractions as well as unfractionated DNA extract (subsampled prior to size selection). PCR was performed in duplicate 25 μl reactions containing: 1 × Quantabio 5Prime HotMasterMix (Quantabio, Beverly, MA, USA), 0.2 μM each of V4/V5 515F-Y and 926R primers [25], 1 μl of DNA template, and 13 μl of molecular-grade water. Primer sequences were modified to include an Illumina overhang adapter. Negative controls and a 16S rRNA mock community (as in Parada et al. [25]) were included. Initial denaturation was performed at 95 °C for 180 s, followed by 30 cycles of 95 °C for 45 s, 50 °C for 45 s, and 68 °C for 90 s, and a final elongation step at 68 °C for 5 min. Oligonucleotide barcodes were added during a second PCR with the following conditions: 95 °C for 180 s, 8 cycles of 95 °C for 30 s, 55 °C for 30 s, and 72 °C for 30 s, and a final elongation step at 72 °C for 5 min. No amplification was observed in the negative controls in a 2% agarose gel. PCR duplicates received the same oligonucleotide barcodes and were pooled after the second PCR.

The *nifH* gene was amplified from unfractionated DNA in duplicate 25 μl PCR reactions containing: 2.5 μl 10 × ExTaq Buffer (+Mg^2+^), 0.5 μl 10 mM dNTPs (Takara Bio USA, Mountain View, CA, USA), 0.5 μl 2.5 mg/ml bovine serum albumin (New England BioLabs, Ipswich, MA, USA), 0.3 μl ExTaq DNA polymerase Hot-Start version (Takara Bio USA), 0.5 μl each of forward and reverse *nifH* primers (10 μM) described in Mehta et al. [16], 1 μl of DNA template, and 19.2 μl of molecular-grade water. Primer sequences were modified to include an Illumina overhang adapter. One negative control was included using 1 μl of molecular-grade water as template. Initial denaturation was performed at 95 °C for 120 s, followed by 35 cycles of 95 °C for 30 s, 55 °C for 30 s, and 72 °C for 45 s, and a final elongation step at 72 °C for 5 min. Oligonucleotide barcodes were added during a second PCR and duplicates were pooled, as described above.

Barcoded PCR products were purified using an Ampure XP bead clean-up kit (Beckman Coulter) at 0.7 × bead solution, pooled in equal concentrations, purified again at 0.7 × bead solution, and sent to the UC Davis DNA Technologies Core Facility (Davis, CA, USA) for 2 × 250 bp paired-end sequencing on an Illumina MiSeq platform.

### Quality filtering and inferring sequence variants

16S rRNA and *nifH* primer sequences were removed from demultiplexed fastq files using cutadapt (v.1.13; [26]). The following filtering and processing steps were performed using DADA2 (v.1.4.0; [27]). Reads were trimmed to 220 bp and reads containing ambiguous bases or >2 expected sequencing errors were removed. Quality-filtered reads were pooled, amplicon sequence variants (ASVs) inferred, paired-end reads merged, and chimeric sequences removed. Merged paired-end sequences were filtered, keeping those 368–378 bp for 16S rRNA gene and 330–370 bp for *nifH*. The *nifH* ASVs that did not align to the target region were manually discarded. Taxonomy was assigned to 16S rRNA sequences via alignment to SILVA Train Set v132. Singleton, unclassified, and eukaryotic sequences were pruned.

### ^15^N-incorporator identification

16S rRNA gene sequences labeled with ^15^N were identified using the multiple window high-resolution DNA-SIP (MW-HR-SIP) method from the HTSSIP software package (v.1.4.0; [28]) in R (v.3.4.0; [29]). The HTSSIP package implements DESeq2 [30] to test if a given ASV is significantly enriched in abundance in a given buoyant density range between control (here, the unincubated sediment) and treatment (here, the sediment incubated with ^15^N_2_). Data were pre-filtered to prune ASVs that: (1) differed in abundance by 10× between unfractionated control-treatment pairs and (2) did not appear in at least three fractions >1.69 g ml^−1^ in both control and treatment. These criteria filtered taxa whose abundances may have been affected due to bottle effects and/or incubation with either N_2_ or CH_4_. Moderated log_2_-fold-changes were calculated per taxon across four buoyant density windows: 1.7000–1.7150 g ml^−1^, 1.7075–1.7225 g ml^−1^, 1.7150–1.7300 g ml^−1^, and 1.7225–1.7375 g ml^−1^ with a null threshold of 0.25. These windows were narrow enough to detect the small expected buoyant density shifts, while containing at least 3 fractions per sample. Fold changes were tested for statistical significance using one-sided Wald tests and adjusted for multiple hypothesis testing using the Benjamini–Hochberg method to a false detection rate of 0.05.

### Evaluating ^15^N-SIP accuracy

To evaluate SIP accuracy, the percentage of false positives as a function of ^15^N-enrichment of the labeled biomass was estimated using simulations in SIPSim (v.0.1; [31]). Microbial communities for simulated experiments were assembled by downloading 816 complete genomes from the NCBI RefSeq database. Percent taxa shared and percent of rank abundances permuted between control and treatment communities were set to match the values from the sample incubated with argon from the 9–12 cmbsf horizon analyzed here. Default SIPSim settings were used, with the exception of simulating 1000 fragments per genome. Simulated OTU tables were then filtered and analyzed following the same pipeline used for the real samples.

Atom percent of ^15^N-labeled biomass was estimated to be 40.4 at% ^15^N by mass balance, using the previous measurement of 0.422 as the total at% ^15^N of the N pool in this sediment [12] and assuming that: (1) 98.7% of total organic N was from dead biomass and (2) 10% of the community was ^15^N-labeled [32]. Given the potential error in these assumptions, specificity estimates for simulated communities with an at% labeling within a factor of two of our ^15^N-labeled biomass estimate (i.e., 20–50 at% ^15^N) were averaged together for the final specificity estimate.

### Closest characterized relatives of ^15^N-incorporators

Closest characterized relatives of ^15^N-incorporators were identified via BLAST search using the NCBI 16S ribosomal RNA sequences database. Complete or near-complete 16S rRNA gene sequences were downloaded, aligned using MAFFT (v.7.055b; [33]), and maximum likelihood phylogenetic trees inferred using RAxML (v.7.7.2; [34]) with the GTR + G + I evolutionary model and 100 bootstrap replicates. One 16S rRNA gene copy from *Methanocaldococcus jannaschii* was used as the outgroup (GenBank accession no. NR_113292).

### Inferring *nifH* host identity

A reference *nifH* database was curated from the NCBI Nucleotide database that contained all sequences annotated as “nifH”, “nitrogenase reductase”, “nitrogenase iron protein”, “anfH”, or “vnfH” (*n* = 83 318 as of August 28, 2018). The database was filtered to remove sequences that were: (1) from unidentified organisms, (2) <200 bp or >1 kb in length, and (3) not flanked by start and stop codons. The filtered *nifH* database (*n* = 6040 sequences) was aligned using MAFFT (v.7.055b), adjusting for sequence direction, and then a phylogenetic tree was inferred using RAxML (v.8.2.12) with the GTR + CAT evolutionary model and 100 bootstrap replicates.

SEPP (v.4.3.5; [35]) was used to insert the *nifH* ASVs into the full-length *nifH* reference alignment and reference tree. After placement on the reference tree, the taxonomic classifications for reference sequences that were within a patristic distance <0.48 (i.e., sum of branch lengths; *k*) to each ASV were obtained using myTAI (v.0.8.0; [36]). Reference sequences within this threshold were 81% (±2.6%) identical to amplicon sequences (min. 71% sequence identity) (Supplementary Fig. S1). The host identity for each *nifH* ASV was then inferred based on these references’ conserved taxonomic ranks. ASVs separated by patristic distances >0.48 to their nearest neighbors remained unclassified. The *k* value was set empirically to the value that maximized the number of annotated ASVs (Supplementary Fig. S2). This balances: (1) between being too restrictive and failing to identify reference sequences near an ASV (<<*k*) and (2) overexploring tree space and including reference sequences from different taxonomic lineages that display patterns of vertical inheritance (>>*k*). All trees were visualized using the Interactive Tree of Life [37].

The sequence placements for the 42 *nifH* ASVs with *k* > 0.48 were inspected manually. Nine of these ASVs were found to display clustering characteristics consistent with a valid taxonomic signature. Eight unidentified *nifH* ASVs clustered with the single *Acidobacteria nifH* sequence available from GenBank, but were not assigned to the *Acidobacteria* due to their patristic distance to *Desulfovibrio nifH* sequences being within the selected *k* threshold (*k*_*Ac-Dv* _= 0.40; *k* = 0.48). However, the failure to classify is likely a function of the poor representation of *Acidobacteria nifH* sequences in the database (*n* = 1); if more reference sequences were available, these ASVs may have been assigned to *Acidobacteria* hosts. Conversely, one ASV clustered within the *Betaproteobacteria*, but its patristic distance to its nearest neighbor was greater than our selected annotation threshold, so it remained unclassified (k_*near* _= 0.49; *k* = 0.48). Thus, while these ASVs were not annotated under the optimized *k* threshold, they are considered to belong to the lineages in their respective clusters.

## Results and discussion

### Identity and relative abundance of ^15^N-incorporators

**Fig. 2:**
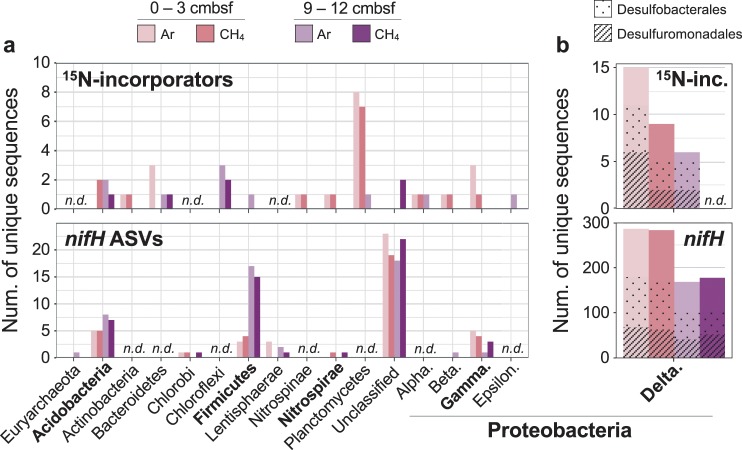
Taxonomic diversity of ^15^N-incorporators and *nifH*-containing taxa identified via ^15^N-SIP and DNA sequencing, respectively. **a** Taxa listed by class for *Proteobacteria* and phylum for all other groups. **b** *Deltaproteobacteria* ^15^N-incorporators and *nifH* ASVs shown separately to accommodate for different vertical axis scales. The fraction of sequences identified as *Desulfobacterales* (dots), *Desulfuromonadales* (stripes), and other *Deltaproteobacteria* (no pattern) is additionally indicated. *n*.*d*. = not detected.

We used ^15^N-SIP to identify organisms that assimilated ^15^N_2_ in our sediment incubations. ^15^N-SIP links taxonomic identity to active function without requiring cultivation or lineage-specific hypotheses [38], thereby increasing the potential to observe a broad community of diazotrophs, including novel taxa [24, 39]. We identified a total of 61 taxa (unique 16S rRNA gene ASVs) as having incorporated ^15^N into their DNA across both sediment depths and headspace compositions (Fig. 2). We hereafter refer to these taxa as ^15^N-incorporators. *Deltaproteobacteria* composed 21 of the 61 ^15^N-incorporators, with the *Desulfobacterales* and *Desulfuromonadales* each containing eight. Outside the *Deltaproteobacteria*, the ^15^N-incorporators were phylogenetically diverse, falling within the *Acidobacteria*, *Actinobacteria*, *Bacteroidetes*, *Chloroflexi*, *Firmicutes*, *Nitrospinae*, *Nitrospirae*, *Planctomycetes*, and *Proteobacteria* (*Alpha*-, *Beta*-, *Gamma*-, and *Epsilon*-).

**Fig. 3:**
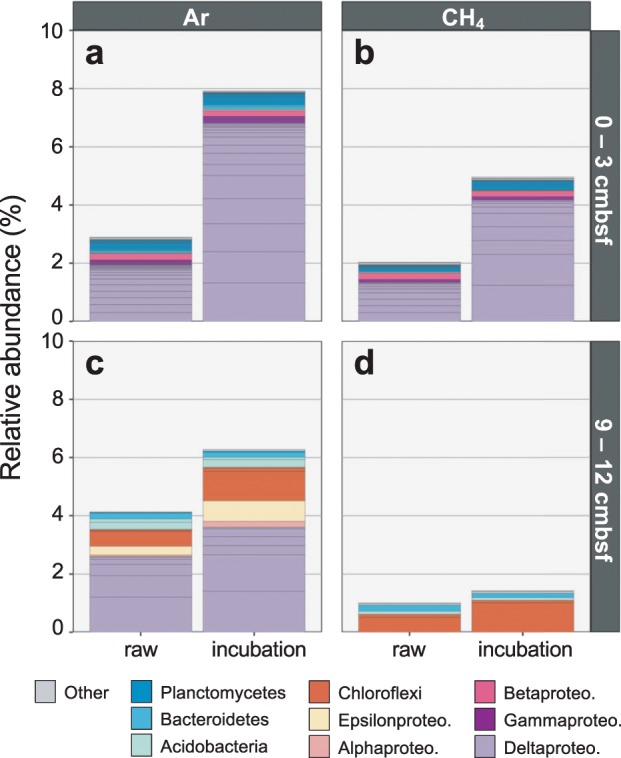
Relative abundance of ^15^N-incorporators in the incubation in which they were identified, as well as the corresponding raw (unincubated) sediment. Relative abundances shown for 0–3 cmbsf samples incubated with argon **(a)** or methane **(b)** and 9–12 cmbsf samples incubated with argon **(c)** or methane **(d)**. Lineages that accounted for >0.1% of 16S rRNA gene reads in at least one sample are colored by class (for *Proteobacteria* only) or phylum. Low abundance lineages (i.e., <0.1% of reads in each sample) are grouped as ‘Other’. Internal bar lines show relative abundances of individual ASVs.

In the 0–3 cmbsf sediment, we identified 34 and 25 taxa as ^15^N-incorporators after incubation with headspaces of argon or methane, respectively (Fig. 2). Seventeen of these were identified as incorporators in both incubations and belonged to the *Nitrospinae* (*n* = 1), *Nitrospirae* (*n* = 1), *Planctomycetes* (*n* = 4), *Betaproteobacteria* (*n* = 1), *Gammaproteobacteria* (*n* = 1), and *Deltaproteobacteria* (*n* = 9) (Supplementary Fig. S3). The taxa identified as ^15^N-incorporators from the Ar incubation at this depth composed 8% of the incubated community’s 16S rRNA gene reads (Fig. 3a). In the corresponding raw (unincubated) sediment, the same taxa composed 3% of reads, indicating their approximate in situ relative abundance. The taxa identified as ^15^N-incorporators from the CH_4_ incubation at this depth accounted for 5% and 2% of the incubated and raw communities, respectively (Fig. 3b).

Fewer taxa were identified as ^15^N-incorporators in the 9–12 cmbsf incubations, consistent with the lower detected rates of N_2_ fixation in that sediment horizon (Fig. 1b). From these incubations, we identified 16 and 6 taxa as ^15^N-incorporators after incubation with Ar or CH_4_, respectively (Fig. 2). These incubations shared three ^15^N-incorporators, one each from the *Acidobacteria*, *Bacteroidetes*, and *Chloroflexi* (Supplementary Fig. S3). The ^15^N-incorporators from the Ar incubation at this depth accounted for 6% of the incubated community’s 16S rRNA gene reads, and the same taxa composed 4% of reads in the corresponding raw community (Fig. 3c). The taxa identified as ^15^N-incorporators from the CH_4_ incubation at this depth composed 1% of reads from both the incubated and raw samples (Fig. 3d). The results from both sediment depths therefore indicate that active diazotrophs represent a relatively low percentage of the in situ microbial communities (<5%).

^15^N-SIP accuracy depends upon a suite of parameters, including experimental design, number of isotopically-enriched taxa, sequencing depth, and computational method of incorporator detection [31]. Using a recently developed software package to quantify SIP accuracy [31], we estimate our ^15^N-SIP analysis has a false positive detection rate of 18% (±16%) (see “Methods”; Supplementary Fig. S4). However, three additional lines of evidence support the conclusion that ^15^N-labeled taxa are identified accurately: the list of ^15^N-incorporators was not predicted by (1) ASV rank abundance, (2) lineages with the most ASVs, nor (3) ASV abundance ratios between control and treatment unfractionated samples (Supplementary Fig. S5). It is also important to note the possibility of false negatives. For instance, we did not identify *Deltaproteobacteria* ^15^N-incorporators in the 9–12 cmbsf incubation with CH_4_ (Figs. 2b and 3d). Although this could indicate a change in community dynamics with methane, their lack of detection may be an artifact introduced during data pre-filtering to minimize false positives (Supplemental Fig. S4).

Separately, cross-feeding of ^15^N-labeled substrates between diazotrophic and non-diazotrophic organisms can inflate ^15^N-incorporator diversity. While previous nanoscale secondary ion mass spectrometry analyses of individual cells from deep-sea methane seep sediments incubated with ^15^N_2_ did not find significant cross-feeding after six months of incubation [14, 22], a length three times longer than the experiments here, it still remains a potential cause of ^15^N-labeling, especially in the case of physically associated cells. To minimize the impact of these potential sources of error, we supplemented the ^15^N-SIP results with a molecular analysis of *nifH*.

### Inferred diversity and identity of *nifH*-containing taxa

To survey the diversity of taxa potentially capable of fixing nitrogen, which includes those not active under the incubation conditions, we performed amplicon sequencing of the *nifH* gene. We recovered a total of 62 748 reads after quality-filtering, comprising 1026 unique ASVs across all raw and incubated sediment samples from both depths. Of these ASVs, 434 were bona fide *nifH* sequences (hereafter referred to as *nifH* ASVs) and 592 were homologs of *nifH*. This high-throughput approach combined with greater sequence resolution recovered nearly an order of magnitude more unique *nifH* sequences than previous deep-sea studies [12, 19], implying a greater diazotroph diversity in the benthos than previously known.

In addition, to corroborate the ^15^N-SIP results with an independent assessment of diazotroph identity, we applied a novel pipeline to infer taxonomic identity from the *nifH* amplicon sequences. Our approach adopts a phylogenetic framework whereby amplicons are inserted into a reference tree of curated *nifH* sequences. Then, the identities of the amplicons’ source organisms are inferred based on the neighboring sequences according to empirically-defined parameters (see *Methods*). When amplicons are located near reference sequences that differ in taxonomic origin, source organism identity is not inferred. This approach therefore minimizes errors in host identification due to horizontal gene transfer. We hereafter refer to the inferred hosts as *nifH*-containing taxa. Following this approach, we were able to propose identities at the phylum rank for 392 of the 434 recovered *nifH* sequences.

Consistent with the ^15^N-SIP results, we identified a phylogenetically diverse assemblage of *nifH*-containing taxa across all samples, with most belonging to the *Deltaproteobacteria* (84%; 331 of 392 ASVs) (Fig. 2). The *nifH* ASVs from inferred *Deltaproteobacteria* hosts dominated the relative abundance in each sample as well, comprising 81% of *nifH* reads from the raw 0–3 cmbsf horizon and 79% from the 9–12 cmbsf horizon (Supplementary Fig. S6) Of the inferred Deltaproteobacterial *nifH* ASVs, 38% were inferred to belong to the order *Desulfuromonadales*, 23% to the *Desulfobacterales*, 3% to the *Desulfovibrionales*, and 37% to undetermined orders. Within the *Desulfuromonadales*, most ASVs fell into one of three *nifH* clusters: those most closely related to the *nifH* sequence from *Desulfuromusa kysingii* (78–84% nucleotide sequence id), to four *nifH* sequences from the genus *Desulfuromonas* (81–89% nuc. id), or to two *nifH* sequences within the genus *Pelobacter* (81–87% nuc. id). These three *nifH* clusters accounted for ~70% of all *nifH* reads from the raw sediments and 26–45% from the incubated sediments. These findings suggest that, while we inferred diverse *nifH*-containing taxa overall, a few clusters of *nifH*-containing taxa from the *Deltaproteobacteria* composed the majority of the in situ diazotroph community.

**Fig. 4:**
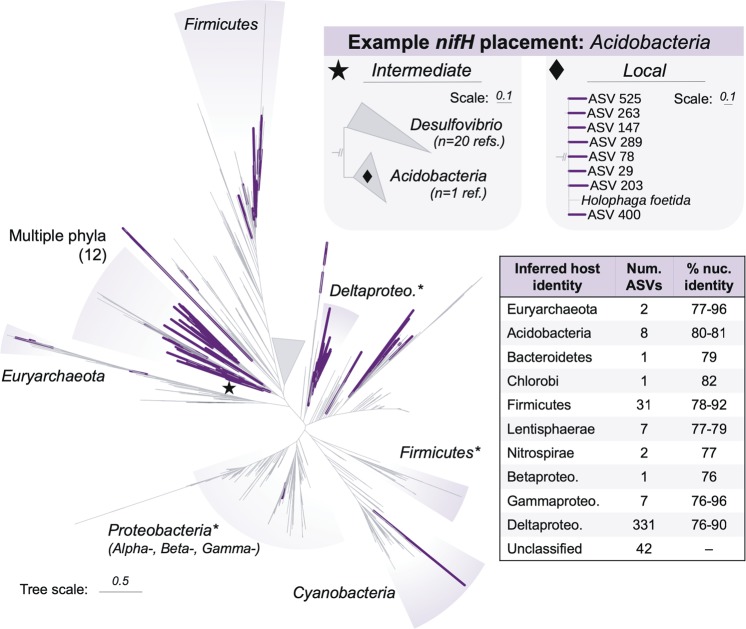
Diversity of recovered *nifH* sequences. Bold branches show *nifH* ASVs (*n* = 434) placed at their maximum likelihood positions on the reference *nifH* tree (*n* = 6040). Shaded regions indicate taxonomic identities of select clades for reference. Gray wedge shows collapsed branches of *nifH* homologs; the full tree including these sequences can be found in Supplementary Fig. S7. Reference sequences on terminal branches with lengths >1.0 were pruned for clarity (*n* = 34). Phyla within clade labeled “Multiple phyla”: *Acidobacteria*, *Actinobacteria*, *Bacteroidetes*, *Chlorobi*, *Chloroflexi*, *Cyanobacteria*, *Elusimicrobia*, *Lentisphaerae*, *Nitrospirae*, *Proteobacteria*, *Spirochaetes*, and *Verrucomicrobia*. Star and diamond insets display example *nifH* ASV placements at finer phylogenetic scales. ‘% nuc. identity’ in table shows range of percent nucleotide identities between *nifH* ASVs and closest references for each taxonomic group. *clades which include one or two *nifH* sequences from other phyla (clades without asterisks contain sequences from one phylum).

Outside the *Deltaproteobacteria*, we recovered many lower abundance *nifH* sequences from phylogenetically diverse inferred taxa, including the *Euryarchaeota* (*n* = 2 ASVs), *Acidobacteria* (*n* = 8 ASVs), *Bacteroidetes* (*n* = 1 ASV), *Chlorobi* (*n* = 1 ASV), *Firmicutes* (*n* = 31 ASVs), *Lentisphaerae* (*n* = 7 ASVs), *Nitrospirae* (*n* = 2 ASVs), *Betaproteobacteria* (*n* = 1 ASV), and *Gammaproteobacteria* (*n* = 7 ASVs) (Fig. 4). Those inferred to be from the *Acidobacteria* and *Firmicutes* were most abundant, and respectively accounted for 6% and 10% of *nifH* reads in the 9–12 cmbsf incubations (Supplementary Fig. S6). The *nifH* ASVs from the *Euryarchaeota*, *Bacteroidetes*, *Chlorobi*, *Lentisphaerae*, *Nitrospirae*, and *Gammaproteobacteria* each accounted for 0.03–3% of *nifH* reads in each sample where detected. With the exception of the *Euryarchaeota* (found in only 9–12 cmbsf), we recovered *nifH* ASVs from each lineage in both 0–3 and 9–12 cmbsf horizons (Fig. 2a). These highly diverse, lower abundance *nifH* sequences suggest a taxonomically diverse assemblage of *nifH*-containing taxa in the deep-sea benthos.

### Comparison of ^15^N-SIP and *nifH* results to identify ‘candidate diazotrophs’

We combine the ^15^N-SIP and *nifH* analyses to present the most convincing cases for diazotrophy, and refer to those taxa as ‘candidate diazotrophs’ if supported by both lines of evidence. Both the ^15^N-SIP and *nifH* results indicate a phylogenetically diverse community of diazotrophs, including the *Acidobacteria*, *Firmicutes*, *Nitrospirae*, *Gammaproteobacteria*, and *Deltaproteobacteria* (Fig. 2). Both datasets also suggest that *Deltaproteobacteria* are the most abundant and diverse group of diazotrophs in both the 0–3 and 9–12 cmbsf sediment horizons. In particular, our combined results suggest that *Deltaproteobacteria* similar to *Pelobacter carbinolicus* and *Desulfuromonas acetoxidans* fixed nitrogen, since: (1) we identify as ^15^N-incorporators 16S rRNA gene ASVs that share >96% nucleotide sequence identity (V4/V5 region) with these two organisms, (2) both organisms contain copies of *nifH* in their reference genomes, and (3) we recovered *nifH* ASVs that clustered with those sequences in all incubations where they were identified as ^15^N-incorporators (81% *nifH* nuc. id for *D. acetoxidans* and 81–89% *nifH* nuc. id for *P. carbinolicus*). These findings provide the first anabolic evidence of active diazotrophy in these two lineages, extending previous molecular studies that implicated them as diazotrophs in both shallow- and deep-sea sediments [18–20, 40, 41].

Although the detected lineages within the *Acidobacteria*, *Firmicutes*, *Nitrospirae*, and *Gammaproteobacteria* each compose <1% of 16S rRNA reads in each sample (Fig. 3), their detection indicates diverse diazotrophs beyond the *Deltaproteobacteria*, and in some cases reveals novel diazotrophs. To our knowledge, this is the first evidence suggesting *Acidobacteria* fix nitrogen in the environment. Diazotrophy within the *Acidobacteria* is supported here by repeated detection of ^15^N incorporation into Acidobacterial 16S rRNA gene ASVs (*n* = 4) and multiple *nifH* ASVs (*n* = 8) (Fig. 2a; Supplementary Fig. S8). The low sequence identity in 16S rRNA gene amplicons between the *Acidobacteria* ASVs and their closest characterized relatives (88–91% nuc. id) suggests these candidate diazotrophs represent novel lineages within this phylum. Currently, a single published *Acidobacteria* genome, *Holophaga foetida* [42], contains the full genetic complement required to fix nitrogen (*nifHDKENB*), but the organism’s ability to fix nitrogen was not assayed at the time of isolation [43]. Although *Acidobacteria* are widespread and can be abundant in sediments (>10% of the bacterial community) [44–47], their ecological functions in marine ecosystems remain largely unexplored.

While the results of the ^15^N-SIP and *nifH* analyses are consistent, 11 taxa were implicated as diazotrophs in only one of the two datasets. We cannot exclude the possibility that these taxa are false positives in a single dataset, and therefore do not refer to them as ‘candidate diazotrophs’ here. However, they may indeed be diazotrophs. Instances where *nifH* amplicons were recovered from a group for which no ^15^N-incorporators were identified (e.g., *Euryarchaeota*) may be due to ^15^N-SIP data filtering (see *Methods*) or may indicate diazotrophic organisms that were inactive or were active but did not fix nitrogen during incubation. Instances where ^15^N-incorporators were identified but lack accompanying *nifH* ASVs (e.g., *Planctomycetes*) may indicate *nifH* primer bias, insufficient sequencing depth, and/or a lack of diazotrophic cultured representatives with available *nifH* sequence data to aid identification. These cases should be further investigated by targeted approaches to assess diaozotrophy, such as fluorescence in situ hybridization coupled to nanoscale secondary ion mass spectrometry.

In particular, the *Planctomycetes* present a compelling case for further investigation. The *Planctomycetes* are a cosmopolitan phylum that lack nitrogen-fixing representatives, though recent metagenomic evidence from the ocean surface indicates that some members contain the full complement of genes required to fix nitrogen [48]. Our ^15^N-SIP results suggest novel members of benthic *Planctomycetes* are active diazotrophs (*n* = 12; 82–94% 16S rRNA nuc. id to closest characterized relatives) (Fig. 2a; Supplementary Fig. S8). Additional work is needed to confirm diazotrophic function within the *Planctomycetes* and help evaluate their ecological role and biogeochemical significance in the marine environment.

### Indications of diverse catabolisms fueling nitrogen fixation

**Fig. 5:**
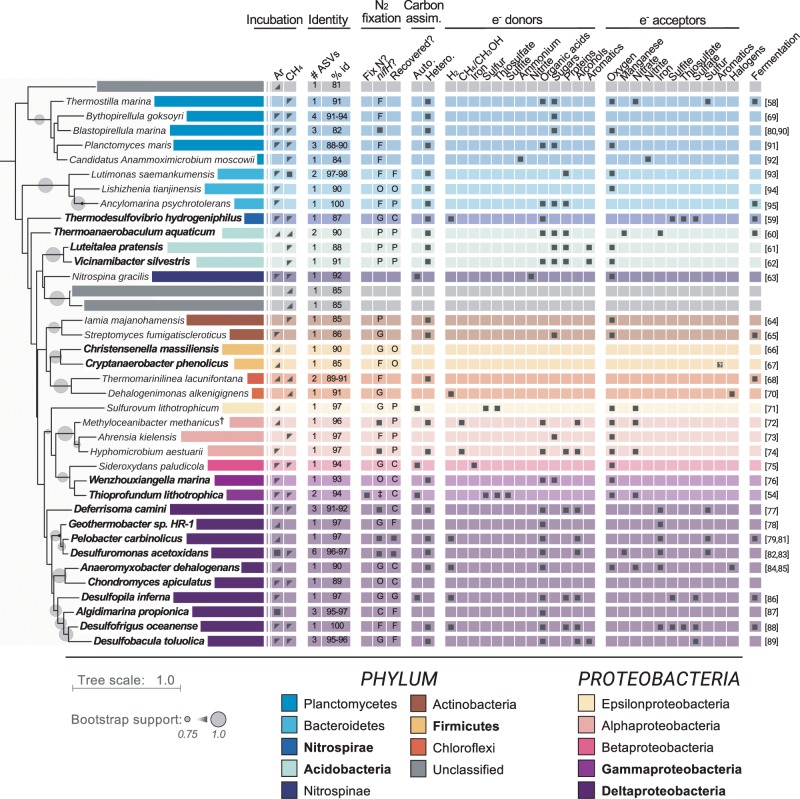
Metabolic profiles of the ^15^N-incorporators’ closest characterized relatives [54, 58–95]. Empty cells indicate metabolisms not tested or not found to support growth. Bold taxon names indicate ^15^N-incorporators within phyla or classes (*Proteobacteria* only) for which *nifH* sequences were also detected. Incubation column: Indicates the headspace (‘Ar’ or ‘CH_4_′) and sediment horizon (0–3 cmbsf, shaded top left corner; 9–12 cmbsf, shaded bottom right corner; both, full shaded square) for which taxa were identified as ^15^N-incorporators. Identity column: ‘# ASVs*’* indicates number of ASVs identified as ^15^N-incorporators that shared the same closest relative; ‘% id’ indicates percent sequence identity to closest relative across the amplified V4/V5 16S rRNA gene region (~370 bp). N_2_ fixation column: ‘Fix N?’ indicates if closest relative previously shown to fix nitrogen (dark square); ‘*nifH*?’ indicates if relative contains a copy of *nifH* in its genome (dark square) or the relative’s lowest taxonomic rank that contains an organism with a copy of *nifH* (P phylum, C class, O Order, F Family, G Genus); ‘Recovered?’ indicates if one of the recovered *nifH* ASV’s closest *nifH* reference sequence was the copy from that relative (dark square) or the lowest common taxonomic rank shared with that relative. Tree rooted to 16S rRNA gene from *Methanocaldococcus jannaschii* (GenBank accession no. NR_113292). †: ^15^N-incorporator shared equal sequence identity to *M. methanicus* and *M. superfactus*. The 16S rRNA gene sequence from *M. methanicus* was used for tree construction and the metabolic profiles for both organisms are shown. ‡: *nifH* sequence not available at the time of analysis.

Previous work has demonstrated that nitrogen fixation at deep-sea methane seeps is dependent on methane and methane oxidizers are the dominant diazotrophs [12, 14, 15, 22]. In the sediments investigated here, ^15^N_2_ assimilation was not dependent on methane (Fig. 1b), suggesting other catabolisms support diazotrophy outside of seeps [12]. Indeed, inspection of the metabolisms associated with each candidate diazotroph’s closest characterized relative revealed diverse potential catabolisms (Fig. 5). We detected five candidate diazotrophs within the *Deltaproteobacteria* whose closest characterized relatives were either obligate or facultative sulfate reducers (95–100% 16S rRNA nuc. id; Fig. 5). This is consistent with previous work demonstrating that sulfate reduction is an important catabolism fueling diazotrophy in shallow marine sediments [7, 9, 20, 49–53]. In addition, closest cultured relatives from 10 of the other candidate diazotrophs have previously been shown to use elemental sulfur as a terminal electron acceptor (91–97% 16S rRNA nuc. id; Fig. 5), further underscoring the likely importance of sulfur metabolism to diazotrophy.

The closest cultured relatives of fifteen of the candidate diazotrophs within the *Acidobacteria* and *Deltaproteobacteria* can use iron as a terminal electron acceptor (90–100% 16S rRNA nuc. id; Fig. 5). This is consistent with recent findings in sediments from the Mauritanian oxygen-deficient zone (47–1108 m water depth), where ferrous iron porewater concentrations, *nifH* gene abundances from *Pelobacter carbinolicus* (an iron- and sulfur-reducer), and nitrogenase activity were found to co-occur [18]. Diazotrophy coupled to iron reduction may therefore be widespread in benthic habitats.

In addition, we recovered six ASVs spanning *Acidobacteria*, *Nitrospirae*, and *Deltaproteobacteria* whose closest characterized relatives can ferment diverse organic substrates (87–100% 16S rRNA nuc. id) (Fig. 5). This is consistent with previous work suggesting that fermentation may support diazotrophy in salt marsh sediments [52]. Interestingly, it suggests that deep-sea nitrogen fixation may be ecologically linked to the remineralization of complex organic matter at both the initial organic hydrolysis (mediated by fermenters) and complete oxidation stages (e.g., oxidation of acetate to CO_2_ by some sulfate reducers).

Strikingly, we identified two candidate diazotrophs from the *Gammaproteobacteria* whose closest cultured relative, isolated from a deep-sea hydrothermal vent chimney [54], is a facultative anaerobe capable of both denitrification and nitrogen fixation (94% 16S rRNA nuc. id) (Fig. 5). We identified two additional ^15^N-incorporators from the *Alphaproteobacteria* and *Epsilonproteobacteria* whose closest cultured relatives are also facultative denitrifiers (97% 16S rRNA nuc. id) (Fig. 5). It is likely that these organisms temporally separate nitrogen fixation and denitrification depending on nitrogen availability. These organisms may therefore alternate between being fixed nitrogen sources and sinks, as has been previously suggested [55, 56], potentially contributing to changes in sediment net fixed nitrogen fluxes [8].

In contrast to methane seep sediments, we have little evidence suggesting that methane oxidation was coupled to nitrogen fixation in these incubations. Although we detected two candidate diazotrophs whose closest relatives can oxidize methane (96–97% 16S rRNA nuc. id), they were identified in the Ar-amended incubations, suggesting they used alternative electron donors during incubation (Fig. 5). Furthermore, the potential catabolisms coupled to nitrogen fixation when methane was added was not overall different from those when methane was not added (Fig. 5). Therefore, while methane cycling coupled to nitrogen fixation in situ remains a possibility, our findings highlight the potential for methane-independent diazotrophy outside of seeps.

Inferring catabolic potential based on comparisons to similar cultured organisms is not definitive, as phylogenetically similar organisms can be metabolically dissimilar. However, other methods to link metabolisms such as culturing and assembling genomes from metagenomes generally assess only a small subset of the community, which makes these approaches insufficient to obtain a community-level perspective. This is particularly true in deep-sea sediments, where the slow growth of microorganisms renders them difficult to obtain in pure culture, and the high intra-species genetic variability presents challenges for assembling high-quality genomes from metagenomes. Therefore, examining the metabolisms of the closest cultured relatives to ^15^N-incorporators, although an indirect assessment of metabolic capacity, provides a broad overview of the diazotrophic community’s potential ecology, which can both inform the design of future studies and aid in their interpretation.

### Implications for deep-sea diazotrophy

Taken together, our findings imply a large diazotrophic niche space in the marine benthos, with the potential for diazotrophic activity across a range of redox and geochemical conditions. Since the samples investigated here were collected at a water depth approaching the average ocean depth and had total organic matter contents (1.2–1.4 wt%) similar to those from continental margins (~1.0 wt%) [57], these diazotrophs may represent those from continental margins more broadly. If indeed widespread, these lineages could impact rates of climatically important metabolisms on the seafloor (e.g., methanogenesis/methanotrophy, organic matter remineralization) by ameliorating ecosystem-level nitrogen limitation. Furthermore, the potential catabolic diversity in the diazotrophic assemblage suggests that benthic diazotrophy may be a stable source of fixed nitrogen despite changing environmental conditions. The catabolic diversity implicated here contrasts with the current understanding of diazotrophs at methane seeps, where nitrogen fixation is methane-dependent and thought to be mediated exclusively by methane-oxidizing archaea and/or methane-dependent sulfate-reducing bacteria [13–15, 18, 22]. Thus, widely distributed, low abundance diazotrophs may serve as a ‘seed bank’ from which certain diazotrophic groups can proliferate if and when environmental conditions select for them, for example, as methane seeps, whale falls, and oxygen minimum zones develop. This taxonomic and metabolic flexibility in the diazotroph assemblage could therefore provide a robust source of fixed nitrogen across vast spatial and temporal scales.

## Conclusions

Our ^15^N-SIP and *nifH* sequencing analyses together suggest that phylogenetically diverse organisms fix nitrogen in deep-sea sediment. The results from both techniques suggest that, on account of their diversity and relative abundance, *Deltaproteobacteria* are important diazotrophs in this habitat, particularly those from the taxonomic orders *Desulfobacterales* and *Desulfuromonadales*. Our findings also suggest that deep-sea sediments host novel diazotrophs of unanticipated phylogenetic breadth, as both datasets detected the *Acidobacteria*, *Firmicutes*, *Nitrospirae*, and *Gammaproteobacteria*, in addition to the *Deltaproteobacteria*. The candidate diazotrophs’ closest cultured relatives can use many terminal electron acceptors, including oxygen, nitrate, iron, sulfur, sulfate, and organic compounds, making it likely that deep-sea diazotrophy is coupled to multiple biogeochemical cycles. These findings have broad biogeochemical implications in considering both modern and ancient environments and highlight the need for additional analyses exploring the diversity and activity of diazotrophs in widely representative marine sediments.

## Supplementary information

Supplemental Material

## Data Availability

All 16S rRNA gene and *nifH* sequence data have been deposited in the GenBank, EMBL, and DDBJ databases under BioProject number PRJEB32101.

## Code availability

Code to reproduce both ^15^N-SIP and *nifH* analyses are available on github (https://github.com/BKapili/SIP_2019).
