# Supplementary material for: Evidence for phylogenetically and catabolically diverse active diazotrophs in deep-sea sediment
Source: ISME J. 2020 Jan 6;14(4):971–83. doi: 10.1038/s41396-019-0584-8 (PMC7082343; doi:10.1038/s41396-019-0584-8)
Supplement: Supplementary file 1 — Supplemental Material [file 41396_2019_584_MOESM1_ESM.pdf]

Supplementary Figures

Evidence for phylogenetically and catabolically diverse active diazotrophs in deep-sea sediment

Bennett J. Kapili[1*], Samuel E. Barnett[2], Daniel H. Buckley[2], and Anne E. Dekas[1*]

[1]Department of Earth System Science, Stanford University, Stanford, CA, 94305
[2]School of Integrative Plant Science, Cornell University, Ithaca, NY, 14850

Email: kapili@stanford.edu, dekas@stanford.edu
[*]Corresponding authors

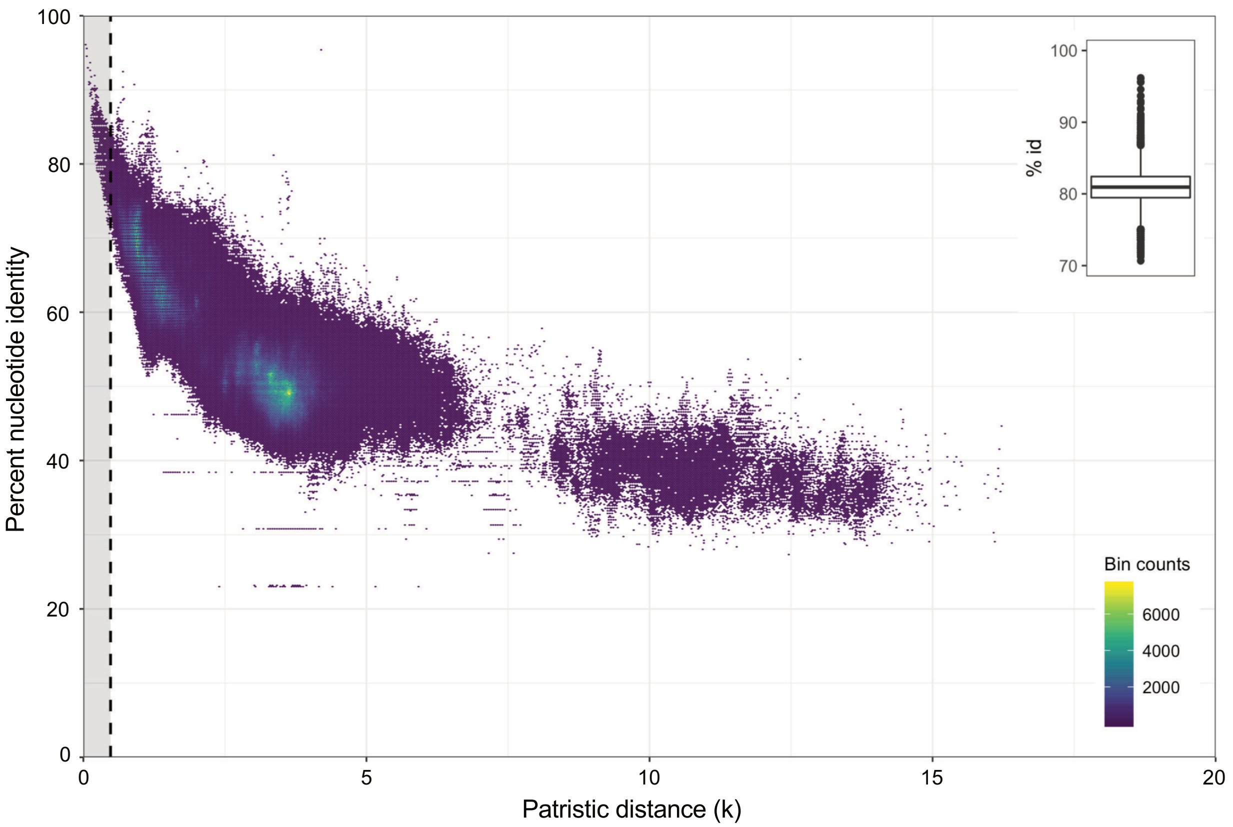

Supplementary Figure S1 Observed relationship between patristic distance (*k*) and percent nucleotide identity for pairwise comparisons between recovered ASVs *(nifH* and homologs) and reference sequences *(*n=6 203 080 total comparisons). Dashed line shows the optimal value of *k* (0.48) for maximizing the number of host inferences given the recovered ASVs. Shaded grey region shows values corresponding to comparisons from which host identities were inferred. Inset shows boxplot of percent identity for pairwise comparisons with $k < 0.48$. Box shows first quartile, median, and third quartile; whiskers show 1.5× interquartile range.

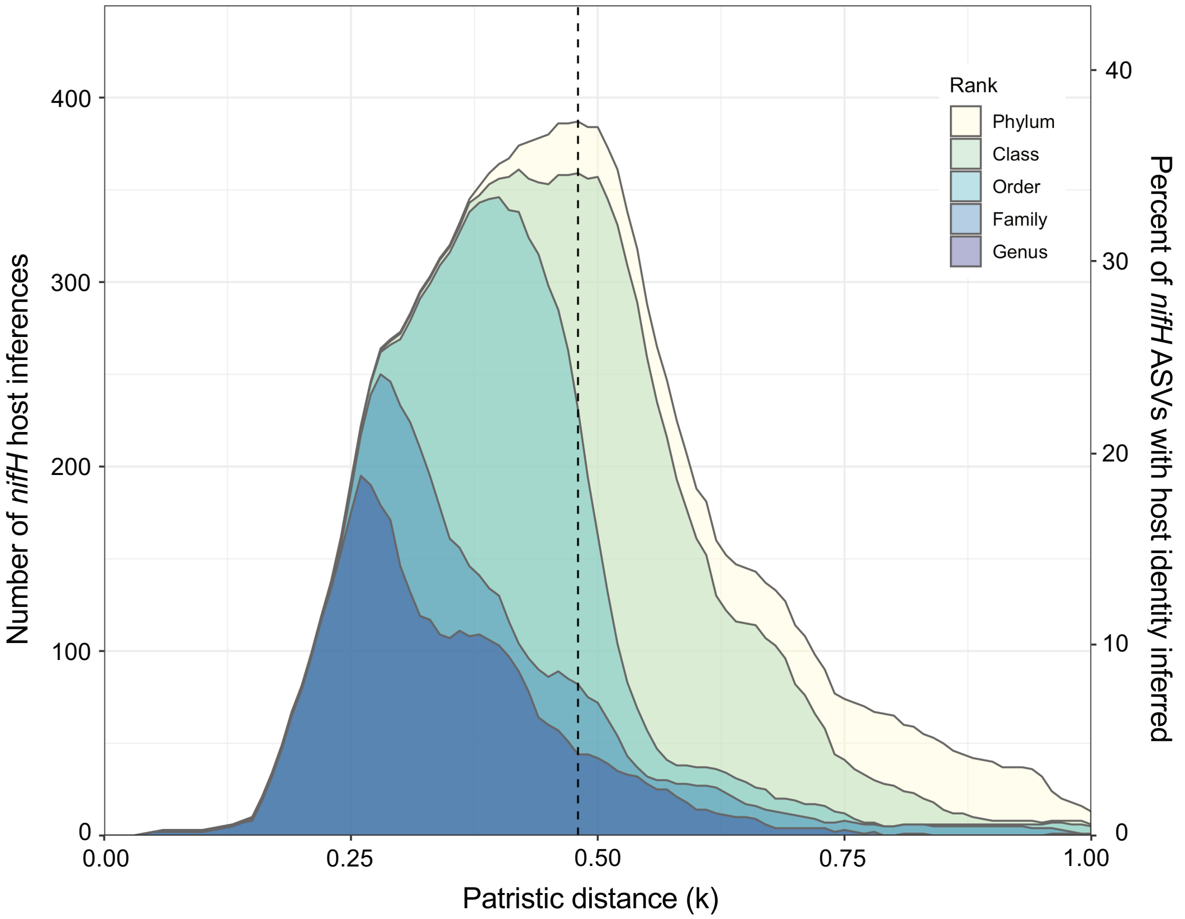

Supplementary Figure S2 Optimal patristic distance (*k*) cutoff for maximizing the number of *nifH* host inferences. Shading corresponds to the number of host identity inferences made for a given taxonomic rank. Dashed line shows the optimal value of *k* (0.48) for the recovered *nifH* ASVs.

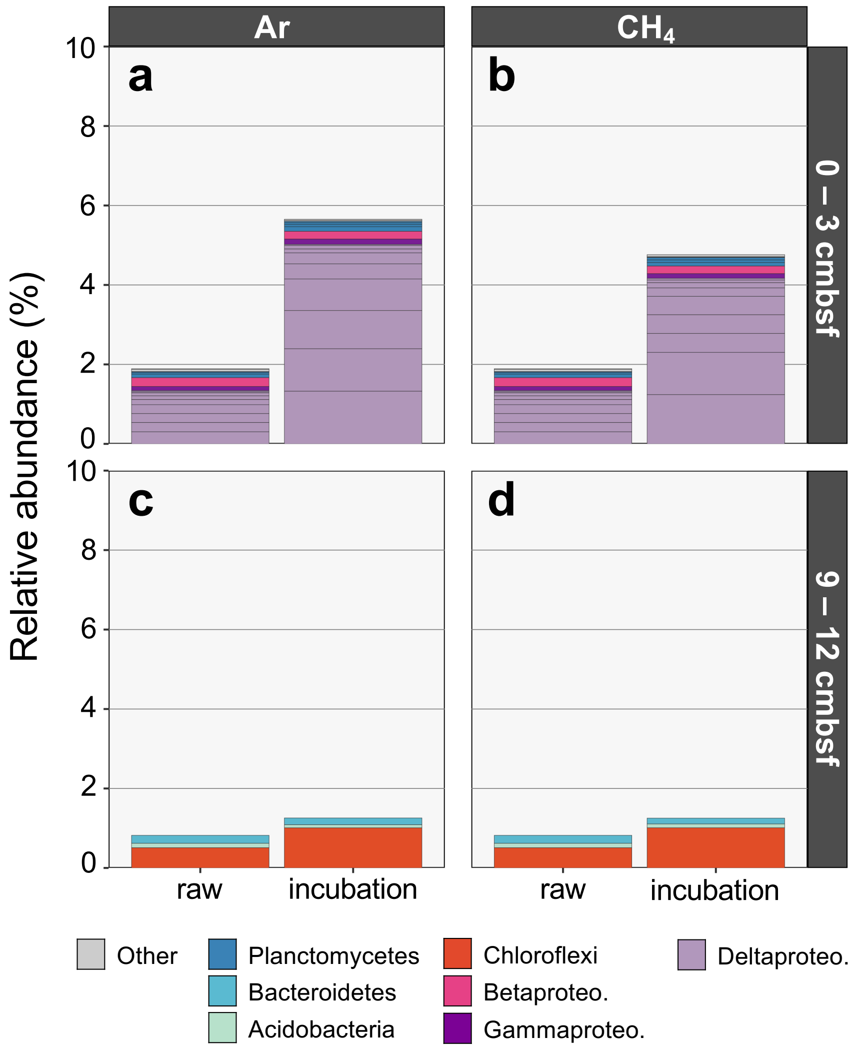

Supplementary Figure S3 Relative abundance of ^{15}N-incorporators shared between both headspace incubations from the same sediment horizon. Values show relative abundances in the incubation in which they were identified, as well as the corresponding raw (unincubated) sediment. **a**–**d** Relative abundances shown for 0–3 cmbsf samples incubated with argon **(a)** or methane **(b)** and 9–12 cmbsf samples incubated with argon **(c)** or methane **(d)**. Lineages that accounted for >0.1% of 16S rRNA gene reads in at least one sample are colored by class (for *Proteobacteria* only) or phylum. Low abundance lineages (*i.e.*, <0.1% of reads in each sample) are grouped as 'Other'. Internal bar lines show relative abundances of individual ASVs.

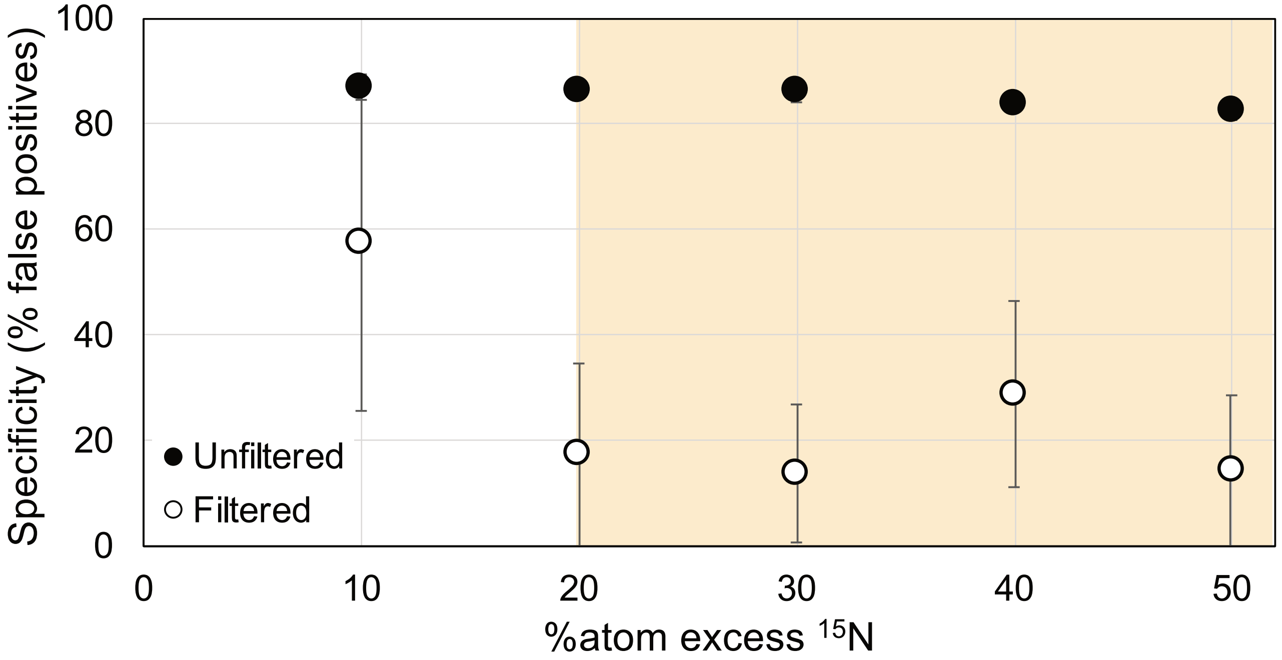

Supplementary Figure S4 Specificity estimates (% false positives) of ^{15}N-SIP analysis as a function of percent label in living biomass for the samples used in this study. Twenty-five experiments were simulated, assigning 10% of taxa to all have an average of 10, 20, 30, 40, or 50 at% ^{15}N-enrichment with ±10% standard deviation in enrichment between taxa and within taxon populations. Five replicate communities were simulated for each at% enrichment. Shaded yellow region shows range of estimates used in final specificity estimate based on the calculated estimate of 40 at% excess in this study (see *Methods*). Black and white dots show specificity estimates for unfiltered and filtered 16S rRNA community data, respectively. Data in this paper were filtered. Error bars show ±1 standard deviation; if none shown, error bars fall within symbol width.

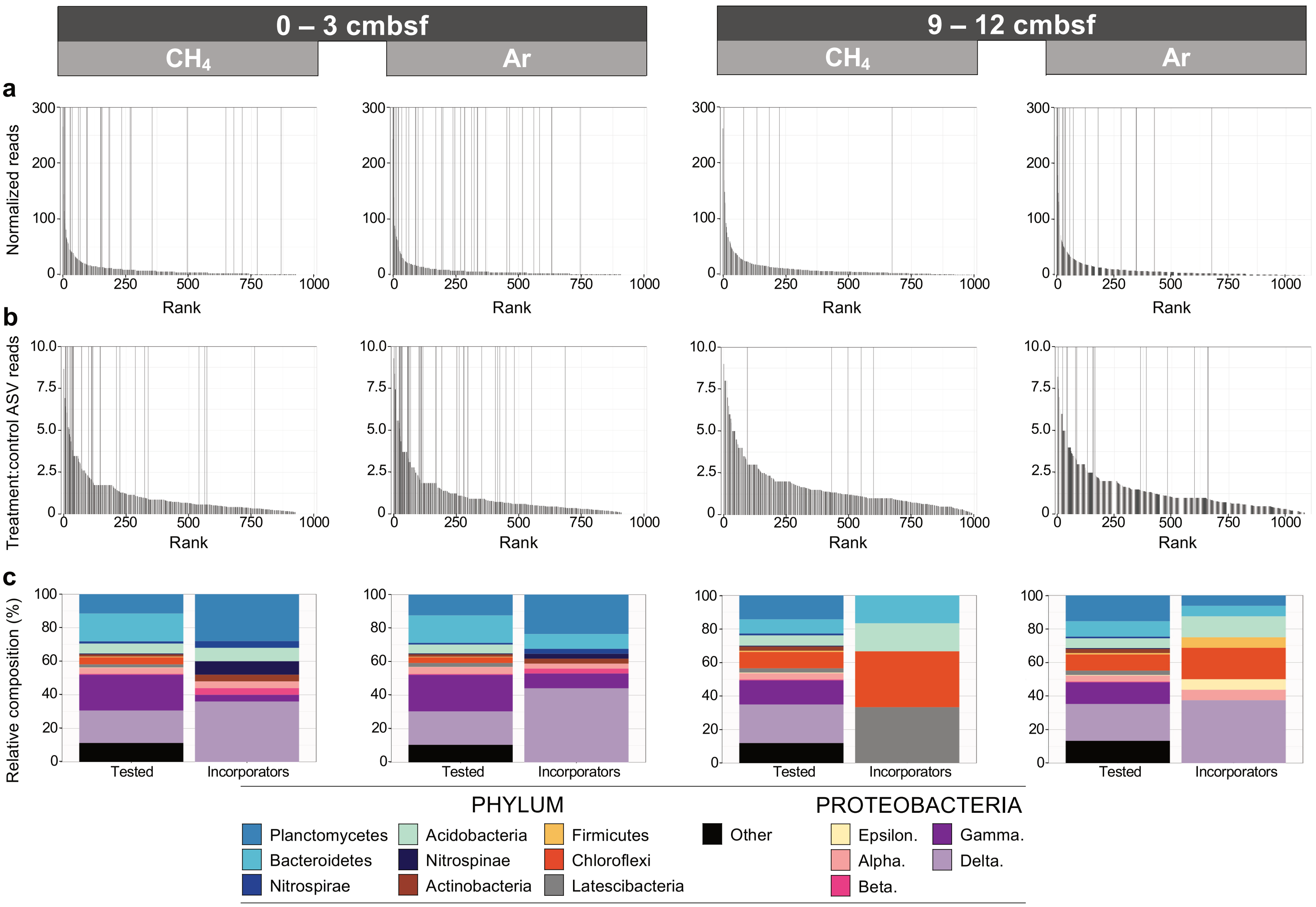

0 – 3 cmbsf
9 – 12 cmbsf
CH_4
Ar
a
Normalized reads
b
Treatment:control ASV reads
c
Relative composition (%)
Rank
Tested
Incorporators
PHYLUM
Planctomycetes
Bacteroidetes
Nitrospirae
Acidobacteria
Nitrospinae
Actinobacteria
Firmicutes
Chloroflexi
Latescibacteria
Other
PROTEOBACTERIA
Epsilon.
Alpha.
Beta.
Gamma.
Delta.

Supplementary Figure S5 Rank abundances of ^{15}N-incorporators and comparisons of community composition between the ASVs tested for ^{15}N incorporation and the ASVs identified as incorporators. Row **a**, Rank abundances of ASV reads in unfractionated treatment samples normalized using median-of-ratios method from DESeq2 (ref. 1). Vertical black lines show ranks corresponding to ASVs identified as ^{15}N-incorporators. Row **b**, Rank abundance ratios of treatment to control ASV reads in unfractionated samples. Vertical black lines show ranks corresponding to ASVs identified as ^{15}N-incorporators. Row **c**, Relative composition of number of taxa per phylum (or class for *Proteobacteria*) tested for ^{15}N incorporation in each treatment and the composition of the identified ^{15}N-incorporators.

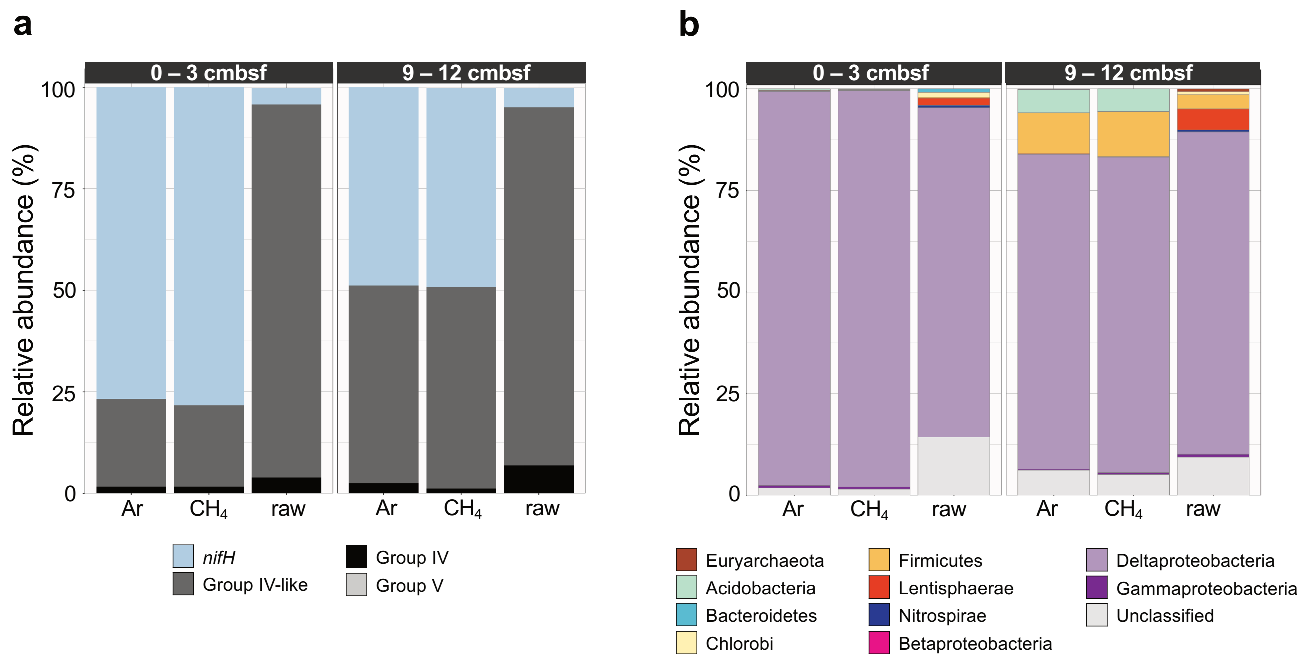

Supplementary Figure S6 Diversity of *nifH* sequences recovered. **a**, Relative abundance of *nifH* and *nifH* homolog (2) ASV reads in each sample. **b,** Relative abundance of inferred *nifH* hosts in each sample. 'Raw' indicates unincubated sediment.

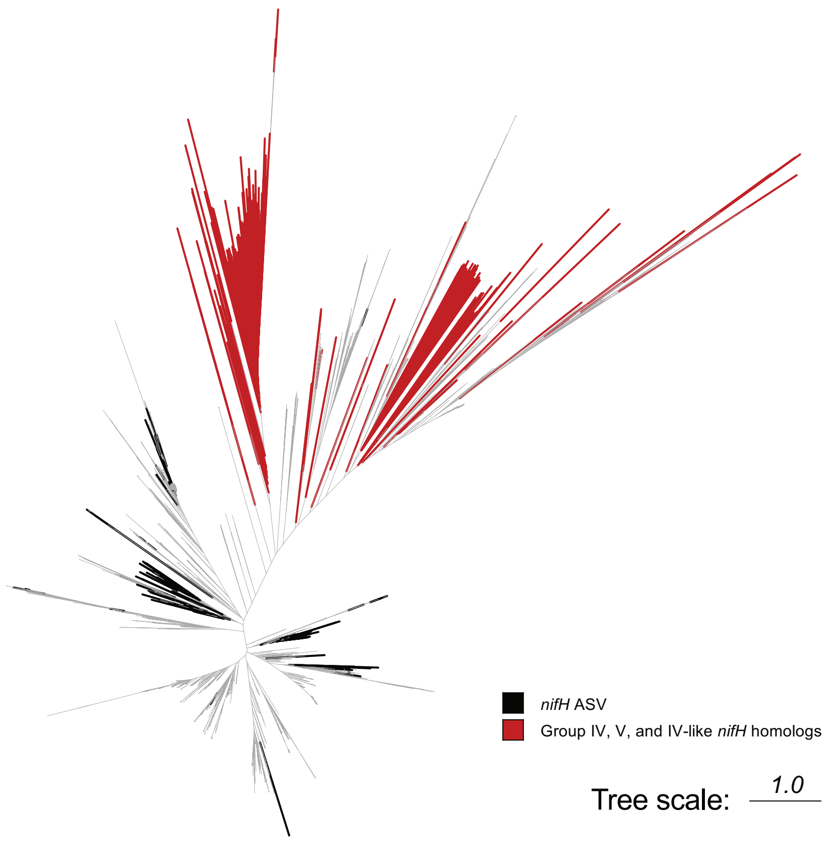

Supplementary Figure S7 Phylogenetic placement of all recovered *nifH* and *nifH* homolog ASVs (n=1026). Bold black branches show *nifH* ASVs, as depicted in Figure 4. Bold red branches show ASVs that clustered with Group IV, Group V, and Group IV-like *nifH* homologs (2).

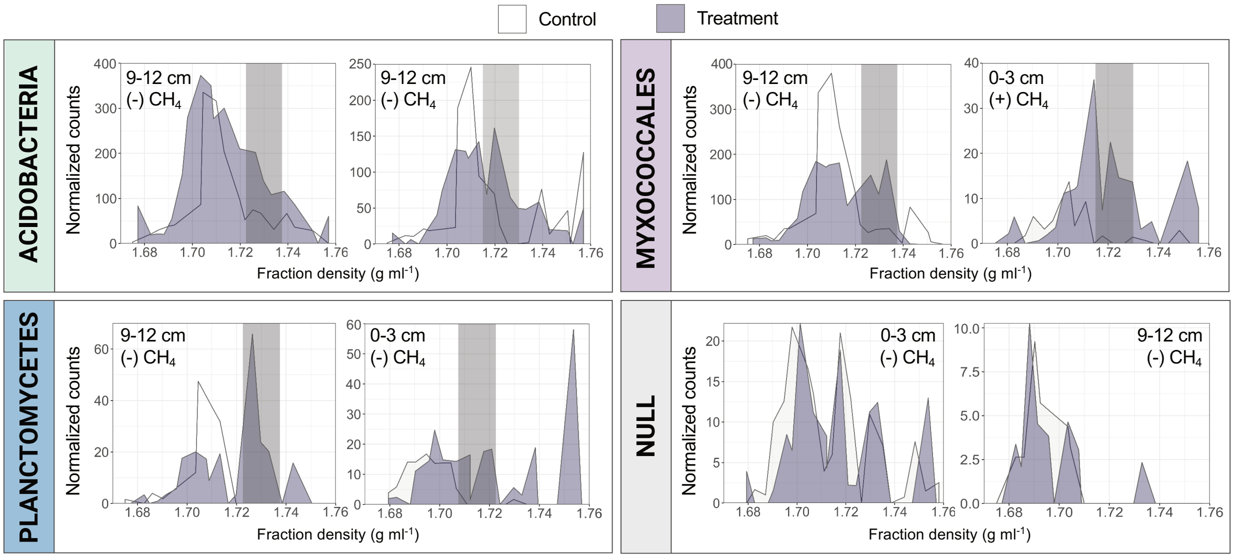

Supplementary Figure S8 Distribution of 16S rRNA reads as a function of ^{15}N-SIP fraction density for selected *Acidobacteria*, *Planctomycetes*, and *Myxococcales* ^{15}N-incorporators. 'Null' panel shows example read distributions for ASVs not identified as ^{15}N-incorporators. Read counts normalized using the median-of-ratios method from DESeq2 (ref. 1). Shaded regions show buoyant density windows for which each ASV was identified as significantly enriched (see *Methods*).

Supplementary References

1. Love MI, Huber W, Anders S. Moderated estimation of fold change and dispersion for RNA-seq data with DESeq2. Genome Biol. 2014; 15(12): 550.

2. Raymond J, Siefert JL, Staples CR, Blankenship RE. The Natural History of Nitrogen Fixation. Mol Biol Evol. 2004; 21(3): 541–54.
